# Defining the Inframammary Fold by Liposuction: An Essential Tool in Aesthetic Shaping of the Reconstructed Breast. Technique and Long-term Results in a Series of Patients

**DOI:** 10.1007/s00266-021-02543-6

**Published:** 2021-08-31

**Authors:** Valentina Pinto, Marco Pignatti, Luca Contu, Riccardo Cipriani

**Affiliations:** 1 Plastic Surgery, IRCCS Azienda Ospedaliero-Universitaria di Bologna, Policlinico di Sant’Orsola, Bologna, Italy; 2 Dipartimento di Medicina Specialistica Diagnostica e Sperimentale (DIMES), University of Bologna, Bologna, Italy

**Keywords:** Inframammary fold, Mammary crease, Breast reconstruction, Liposuction, Breast surgery refinement

## Abstract

**Background:**

A good inframammary fold (IMF) definition and position is essential to achieve a satisfactory and natural result in breast surgery. This structure can be damaged, especially during mastectomies. Multiple methods are reported in the literature to restore IMF or improve its definition. In this study, we present the results achieved in a series of patients treated with subdermal liposuction.

**Methods:**

We report on all our patients who underwent IMF liposuction between January 2016 and June 2020. Subdermal liposuction was performed with a blunt 3 mm cannula along the new IMF to promote skin retraction and adherence between skin and fascia. Results were evaluated subjectively by the patients and objectively by 8 individuals not involved with the treatment.

**Results:**

We performed IMF liposuction in 88 breasts (69 patients), aged 21–74 (mean 52) years for 82 implant-based reconstructions, 2 tuberous breasts, and 4 contralateral breast augmentations. Mean follow-up was 28 months (6–64). Subjective results: the overall result evaluated with the VAS scale reached 86.6/100. All the 22 patients interviewed judged as well defined the new inframammary fold. Objective results: in 83% of cases the definition of the inframammary fold was judged as good or excellent, while symmetry with contralateral IMF, natural appearance, and overall aesthetic outcome were judged as good.

**Conclusion:**

Based on our long-term satisfactory results, we recommend the technique of subdermal liposuction to improve the definition of IMF in breast reconstruction after mastectomy and other breast procedures. It is effective, easy to perform, minimally invasive, and durable.

**Level of Evidence IV:**

This journal requires that authors assign a level of evidence to each submission to which Evidence-Based Medicine rankings are applicable. This excludes Review Articles, Book Reviews, and manuscripts that concern Basic Science, Animal Studies, Cadaver Studies, and Experimental Studies. For a full description of these Evidence-Based Medicine ratings, please refer to the Table of Contents or the online Instructions to Authors www.springer.com/00266.

## Introduction

The inframammary fold (IMF) is an essential aesthetic component of the breast, limiting its lower quadrants and determining the amount of ptosis.

Its presence and definition are of extreme importance to obtain satisfactory, natural results after various kinds of breast surgery [1–3].

Loss of definition of the inframammary fold can occur after mastectomy due to an extensive subcutaneous dissection to remove the most caudal margins of the mammary gland, but even during conservative mastectomy, such as the nipple skin-sparing mastectomy, the inframammary fold is sometimes disrupted.

Without such an important anatomical landmark, a reconstructed breast would look unnatural and disturbingly different from the contralateral, natural one.

Therefore, after mastectomy and sometimes in aesthetic breast surgery, reconstruction or redefinition of the IMF plays a key role in achieving satisfactory results. Several surgical techniques have been described to reach this scope [4].

Knowledge of the anatomical structure of the IMF is crucial to devise a technique for recreating it.

Although earlier studies [5] had suggested that a supporting ligament running from the dermis to the rib cage created the visible skin IMF, Muntan et al., [3], despite an accurate histologic study, could not identify in the fold region any demonstrable ligamentous structure of dense connective tissue. They found, instead, that there is a fused region of adherence between the dermis of the fold and the superficial and deep fascial planes. Bundles of collagen fibres arising from the superficial fascial layer were observed consistently in sections from the sternum to the middle axillary line [3]. Also, Nava et al. [6] on the basis of cadaveric dissections, demonstrated that the IMF is primarily based on deepening of the superficial fascia of the breast that connects to the anterior capsule.

According to this widely accepted interpretation of the IMF anatomy, the adipose component is minimal, even in overweight patients.

A recent review by Kraft et al. [4] has summarized the available techniques and the results published by the authors.

Several techniques proposed for IMF reconstruction aim to fixate skin and dermis to the chest wall. They include stitch positioning, either through an external approach (direct skin incision on the fold) or an internal approach (internal running or single stitch suture accessing to the implant pouch from the mastectomy scar), implant capsule remodelling or local flaps, either dehepitelized skin or adipofascial flaps, external tools, acellular dermal matrix [4].

Mastectomy pattern, breast size and breast shape, skin elasticity, body habitus, and preferences of the surgeon are only a few of the variables that can significantly influence the selection of the surgical approach and the final results of inframammary fold reconstruction. Objective comparison between different techniques, however, is difficult because, as confirmed in the recent review by Kraft mentioned above [4], only many small studies have been published so far.

In 1989, Pinnella [7] suggested that the inframammary crease could be created by liposuction in reconstructed breast lacking this structure. The technique did not have a wide diffusion and no other studies on this method can be found in the literature.

Starting in 2016, we have adopted Pinnella’s modified technique, associating it, in some cases, with fold defining stitches.

In this retrospective study, we describe the technique used in our patients to recreate and define the IMF during different breast surgeries (mainly post-mastectomy reconstruction) and present the long-term results achieved.

## Patients and Methods

We reviewed all the records of the patients operated at our Institution between February 2016 and December 2020 by one of us (VP) for IMF reconstruction using liposuction.

Only the women with at least 6 months of follow-up and willing to participate in the long-term evaluation were included in the study.

The patients gave informed consent to the procedure and to the collection of their data for publication. The Hospital Ethical Committee approved the study (BrREC2020 13/4/2020).

### Surgical Procedure

We describe here the technique used in the most common situation, i.e. IMF asymmetry at the end of expander inflation during post-mastectomy reconstruction. Other indications for the use of this technique are immediate direct-to-implant (DTI) post-mastectomy breast reconstruction, symmetrization procedures on the contralateral breast, exchange of breast implants in previous post-mastectomy reconstruction, remodelling of tuberous breast, and treatment of congenital asymmetries.

**Fig. 1:**
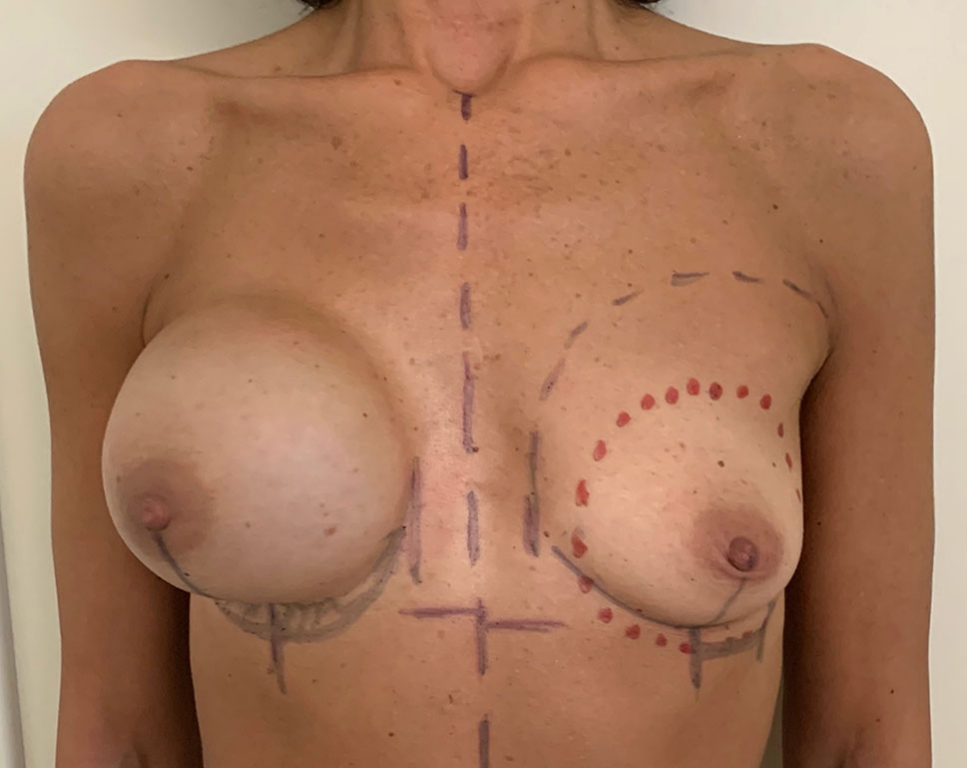
Preoperative markings. The site and curvature of the IMF on the mastectomy side are marked symmetrically to the contralateral breast. In this case, the exchange of the expander with an implant is planned on the right breast, while an augmentation of the left breast is used to improve symmetry. Therefore, the markings of the new IMF on the left are at a more caudal position compared to the natural IMF. Using this as a reference, the new planned IMF of the right side is also marked more caudal than the existing one

With the patient in a standing position, we note and mark the site and curvature of the IMF on the non-operated breast, and we then specularly transpose it to the side of the reconstructed breast (Fig. 1).

The IMF reconstruction is performed at the time of exchanging the expander with an implant, under general anaesthesia with complete muscle relaxation. The patient is placed in a supine position with both arms adducted to the trunk, to avoid altering the IMF position.

If other procedures are performed in the axillary area, both arms are abducted, symmetrically, at 90°, but prepped to be adducted later on during the operation to reproduce the natural position of the IMF previously marked while the patient was standing.

A surgical bed that allows a sitting position during the surgical procedure is needed.

After implant positioning and deep suturing of the surgical wound, the patient is seated and the IMF liposuction is started without the preparatory subcutaneous infiltration usually used in liposuction, to avoid morphologic distortion of the crease.

**Fig. 2:**
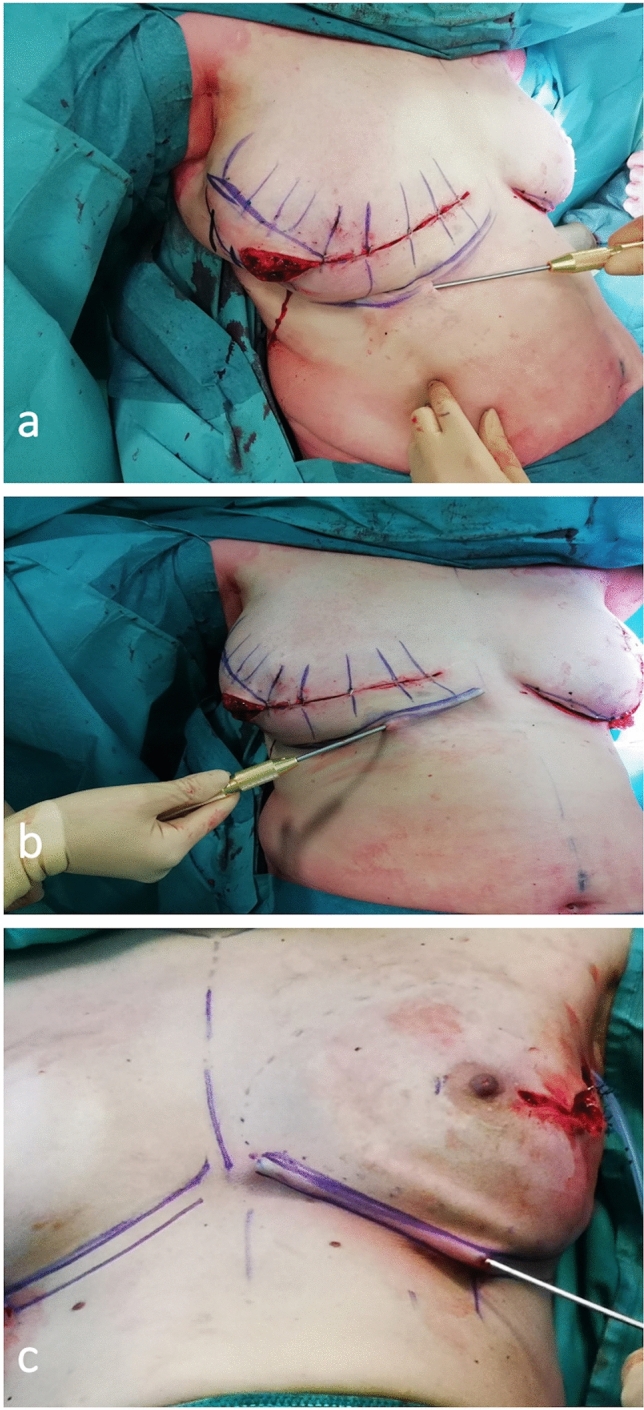
**a** Blunt 3 mm cannula of 15 cm in length is introduced through an horizontal 3 mm skin incision at the mid-point of the planned new IMF. Liposuction of the lateral half of IMF. **b** Liposuction of the medial half of IMF. **c** Another example of liposuction on a direct-to-implant reconstruction after a nipple skin-sparing mastectomy. Note the subdermal position of the cannula

A blunt 3 mm cannula of 15 cm in length is introduced through a horizontal 3 mm skin incision at the mid-point of the planned new IMF (Fig. 2a).

We perform liposuction with a SuperDry technique, in a subdermal plane, along the medial and lateral halves of the IMF (Fig. 2b), moving the holes of the cannula towards both the deep layer and the dermis to promote skin retraction and subsequent tenacious adherence between the skin and the fascia (Fig. 2c).

A short cannula can be more precise in controlling the exact position of the aspirating tip and therefore the correct site of the subsequent subcutaneous scarring and skin retraction. Occasionally, we prefer to use an even shorter (10cm) and smaller (14G) cannula, such as the Coleman fat harvesting cannula that can be curved when necessary.

The area treated with liposuction should be narrow (1 cm approximately) and following exactly the preoperative markings to better define the new IMF position and avoid excessive disruption of the area.

The shape, size, and symmetry of the breasts and inframammary folds are checked again, and if needed, additional liposuction is performed.

When, less frequently, the preoperative position of the expander is too high and the IMF is completely missing or skin expansion is insufficient to accommodate an implant of the right size, we perform a supplementary manoeuvre in addition to the IMF liposuction.

With an inverted abdominoplasty, dissecting for several centimetres in a caudal direction, over the deep fascia plane, we recruit skin from the abdomen and fix it to the chest wall with four to six stitches that, therefore, becomes skin of the lower pole of the reconstructed breast. These stitches are meticulously positioned along the newly desired IMF, mirroring the contralateral one [8–10].

After planning their position by transfixing the skin with hypodermal IV needles of 23 Ga, non-absorbable, monofilament, 2-0 stitches are positioned into the dermis, without piercing the skin, and secured to the chest wall to recreate adequate breast ptosis while leaving a sufficient skin envelope for the implant of the appropriate size. Usually, 4 or 5 such stitches are needed to recreate the natural length and curvature of the new IMF.

A dimple in the skin that will disappear in the following weeks is often visible from the outside where the stitches are positioned.

The sizer is introduced, the patient is seated and the mammary crease position is checked and corrected if needed with additional or differently positioned stitches. At this point, we introduce the final implant, close the wound and perform the liposuction of the IMF a few millimetres caudal to the dermal dimple of the subcutaneous stitches, until adequate definition of the IMF is obtained.

**Fig. 3:**
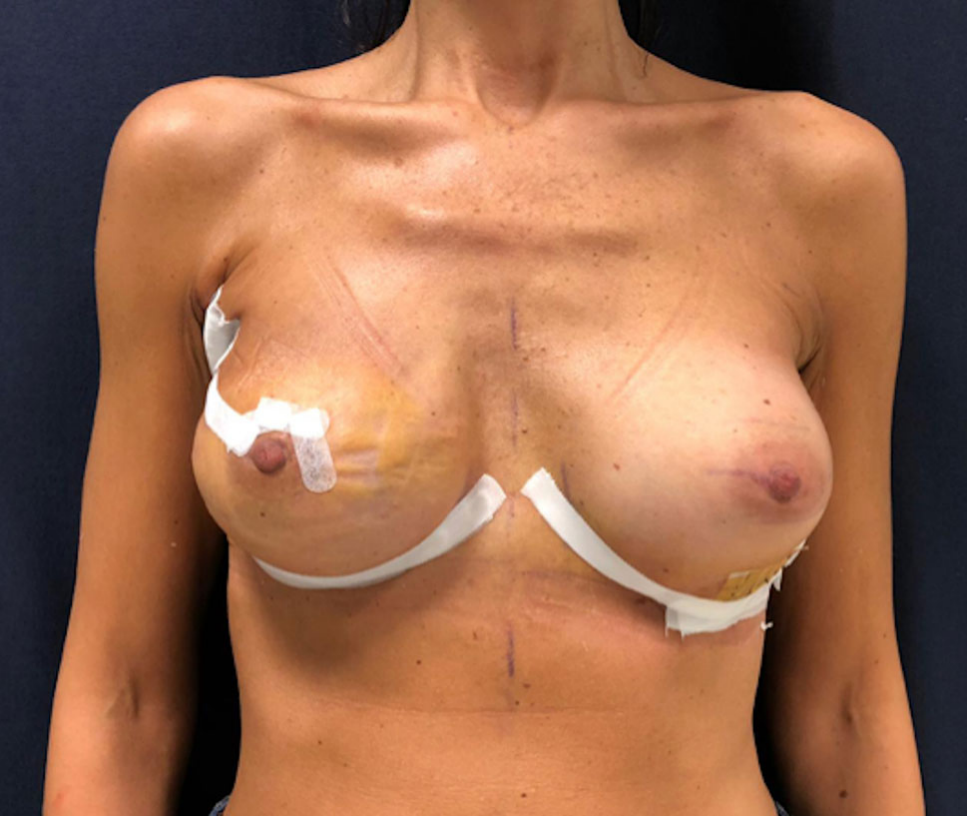
Same patient of Figure 1 two weeks post-operatively with the silk tape dressings still in place

We then like to apply a 10-mm-wide silk tape, under traction, from medial to lateral over the preoperative markings of the new IMF (Fig. 3).

Two layers of tape may be applied, if needed, to produce adequate compression.

Maintaining the silk tape in situ for 4–6 weeks is essential to promote skin retraction and scarring between the tissue layers previously treated with the suction cannula and, in the end, to reproduce the anatomy of the normal IMF.

A tied-over compressive dressing is useful to reduce swelling and prevent hematoma formation.

Four or five days after surgery, the compressive dressing is removed, leaving the silk tape on site, and the patient is provided with a post-operative bra (with compressive band at the breast upper pole) to be worn day and night for at least 4 weeks. The bra will apply a continuous downward pressure that optimizes the implant position and the breast ptosis over the newly created or redefined IMF.

The described technique can be used to make the IMF more symmetrical and more defined also during direct-to-implant breast reconstruction, when performing surgery on the contralateral breast, or when treating tuberous breasts and breast asymmetries.

### Result Assessment

Results were assessed both subjectively and objectively.

Twenty-two patients recorded subjective results that included satisfaction on the overall result evaluated with the VAS scale, patient’s opinion on the definition of the IMF (defined/not defined), and patient’s report of complications.

Objective results included complications, collected by reviewing the institutional database, and aesthetic outcomes.

To objectively assess the aesthetic results, we asked the opinion of two groups of individuals (two females and two males from the health personnel not involved with the treatment, and two females and two males from the administrative personnel) to whom we showed the post-treatment photographs of 20 randomly chosen patients.

We asked them to judge as either bad, suboptimal, good, or excellent the definition of the IMF, the natural appearance of the reconstructed breast, the symmetry compared to the contralateral breast, and the overall aesthetic outcome.

## Results

We reconstructed the IMF in 88 breasts of 69 patients.

In detail, 82 were breast reconstructions with implants (20 direct-to-implant reconstruction, 56 expander–implant, and 6 implant–implant), 2 were tuberous breasts, and 4 were contralateral breast augmentations with implant. Table 1 reports the patients’ demographics.

**Table 1:** Demographic and clinical data of the patients

Demographic and pathological data		
*Total breasts *	*n* = 88	
Monolateral	*n* = 50	56.8%
Bilateral	*n* = 38	43.2%
*Total patients *	*n* = 69	
Average age	52 yrs	(range 21–74)
*Pattern of breast surgery*		
R-m	*n* = 53	60.2%
NSS-m	*n* = 29	32.9%
Contralateral augmentation	*n* = 4	4.3%
Tuberous breast	*n* = 2	2.3%
*Type of surgery*		
EXP/IMPL	*n* = 53	60.2%
IMPL/IMPL	*n* = 6	6.8%
DTI	*n* = 29	22.7%
BA	*n* = 6	6.8%
LD EXP/IMPL	*n* = 3	3.4%
*Associated conditions*		
RT	*n* = 13 12/13 RT before IMF liposuction 1/13 RT after IMF liposuction	14.8%
CC	*n* = 1	1.1%
CC + RT	*n* = 2	2.3%
Br IR	*n* = 1	1.1%

R-m = Radical mastectomy; NSS-m = nipple skin-sparing mastectomy; EXP/IMPL = expander/implant; IMPL/IMPL = breast implant exchange; DTI = direct to implant; BA = (submuscular) breast augmentation; LD = latissimus dorsi; RT = radiotherapy; CC = capsular contracture; Br IR = breast implant rupture

We used the double technique, adding the stitches to liposuction, in 12 breasts out of 88, in which the preoperative position of the IMF was very asymmetrical compared to the non-operated side, or dislocated compared to normal. In the patients requiring the addition of stitches, either the breast expander or implant were incorrectly positioned, being too cranial, or the shape of the curvature of the IMF was too different from the contralateral to be modified with liposuction alone.

Ecchymoses developed in all the patients and resolved spontaneously in 7–10 days. We observed no major complications, skin necrosis, infection, hematoma, or excessive pain.

No surgical revision to redefine the IMF for loss of definition was needed in any of our patients.

**Fig. 4:**
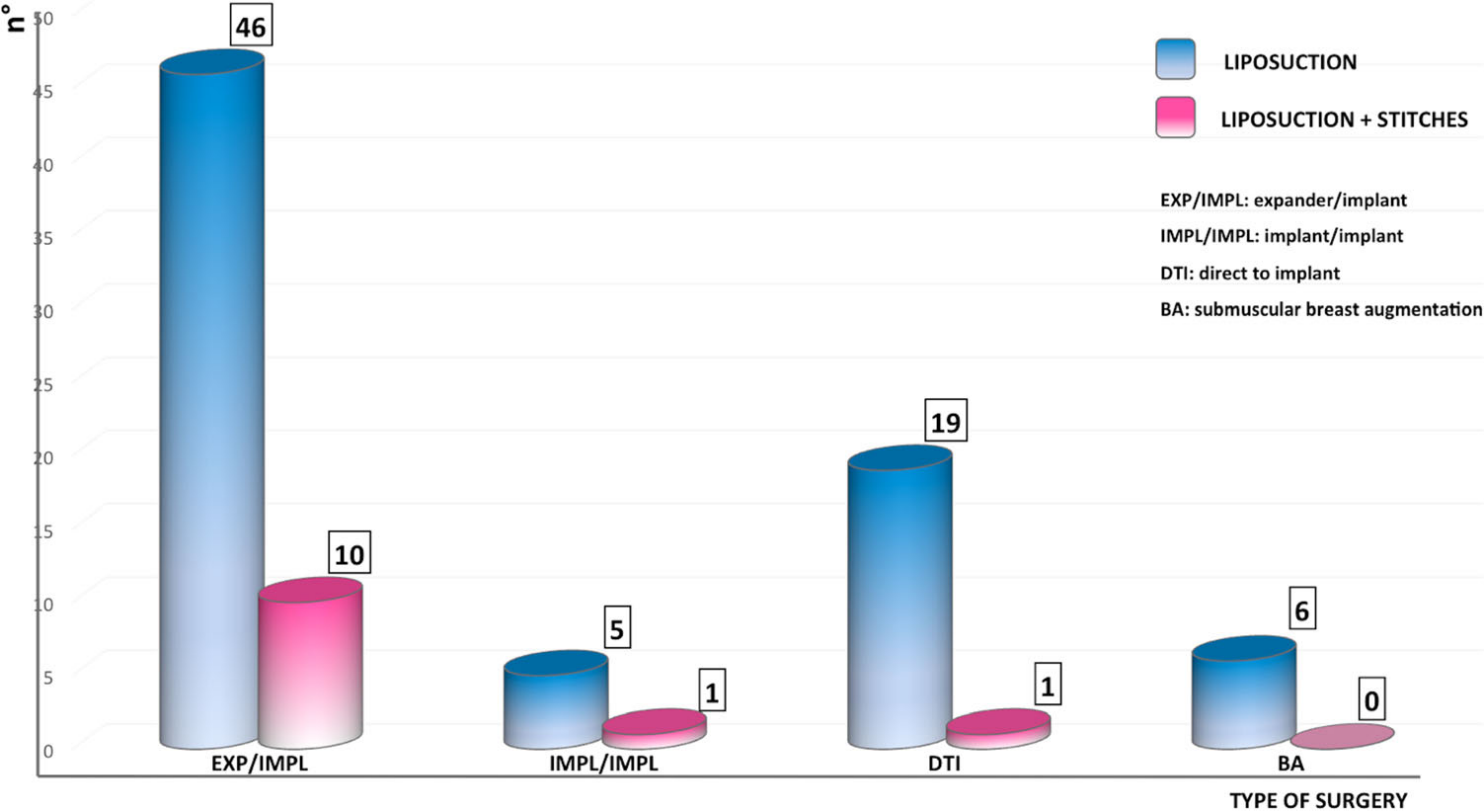
The columns show the different breast surgeries in which the IMF dermal liposuction technique was used (liposuction alone in blue columns, in conjunction with stitches positioning in pink columns). Legenda. EXP/IMPL: Exchange of expander with implant. IMPL/IMPL: Exchange of a ruptured implant with new implant (for reconstruction). DTI: Direct-to-implant reconstruction. BA: Breast augmentation (subpectoral dual plane), either for the treatment of the contralateral breast in post-mastectomy reconstruction (4 breasts) or in tuberous breast deformity (2 breasts)

Figure 4 shows the surgical techniques used.

### Subjective Results

The patients expressed their opinion on the overall result with the VAS system. The mean value obtained was 86.6/100.

All the patients interviewed (22/22) judged as well defined the inframammary fold.

No loss of definition developed over time.

No patient reported complications in the post-operative period.

### Objective Results

**Fig. 5:**
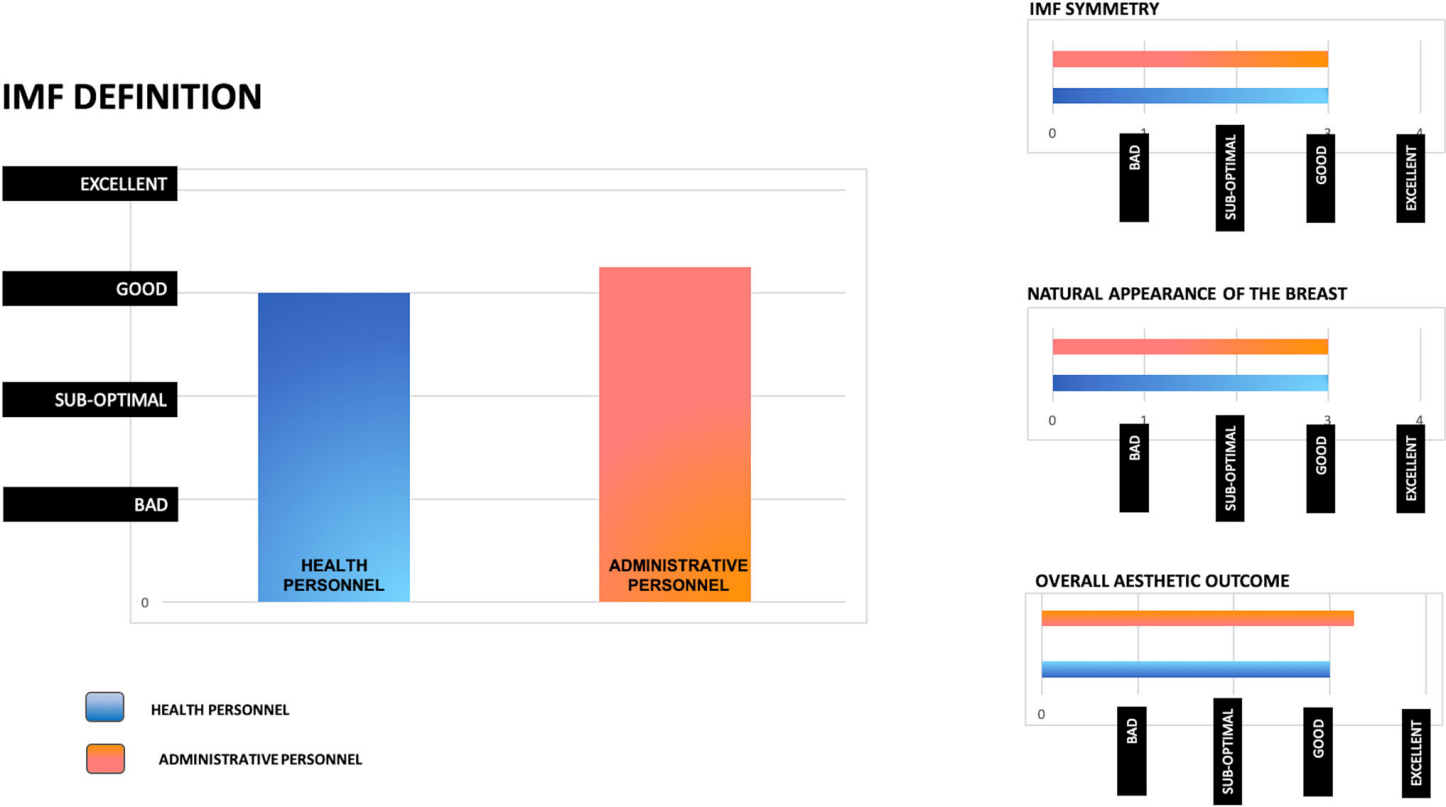
Objective results of surgery as judged by eight evaluators (four chosen among health personnel and four from non-health hospital personnel) as either bad, suboptimal, good, excellent. Left: Degree of IMF’s definition obtained. Right above: Symmetry of IMF. Right middle: Natural appearance of the reconstructed breast. Right below: Overall aesthetic outcome

Figure 5 reports the objective results relative to the degree of IMF definition obtained.

The eight individuals that were invited to judge the results of 20 random patients considered the definition of the inframammary fold as good or excellent in 133/160 answers.

All of them judged as good the natural appearance of the reconstructed breast, the symmetry compared to the contralateral breast, and the overall aesthetic outcome.

**Fig. 6:**
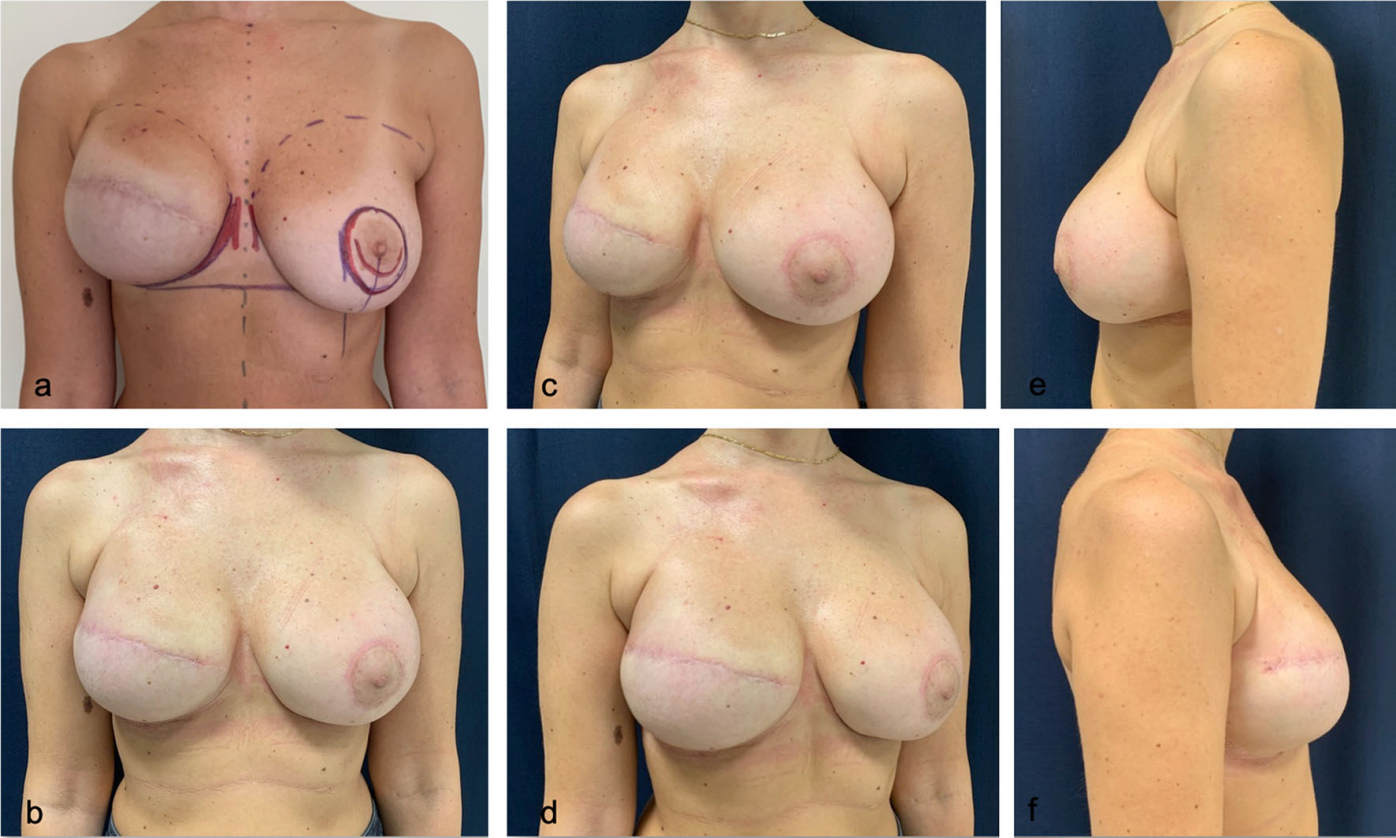
An example of fine-tuning of IMF definition starting from an already acceptable preoperative situation. Preoperative markings (**a**) and post-operative results (**b**, **c**, **d**, **e**, **f**) of a patient who underwent IMF’s liposuction on the right reconstructed breast during exchange of expander with implant and contralateral periareolar mastopexy augmentation

**Fig. 7:**
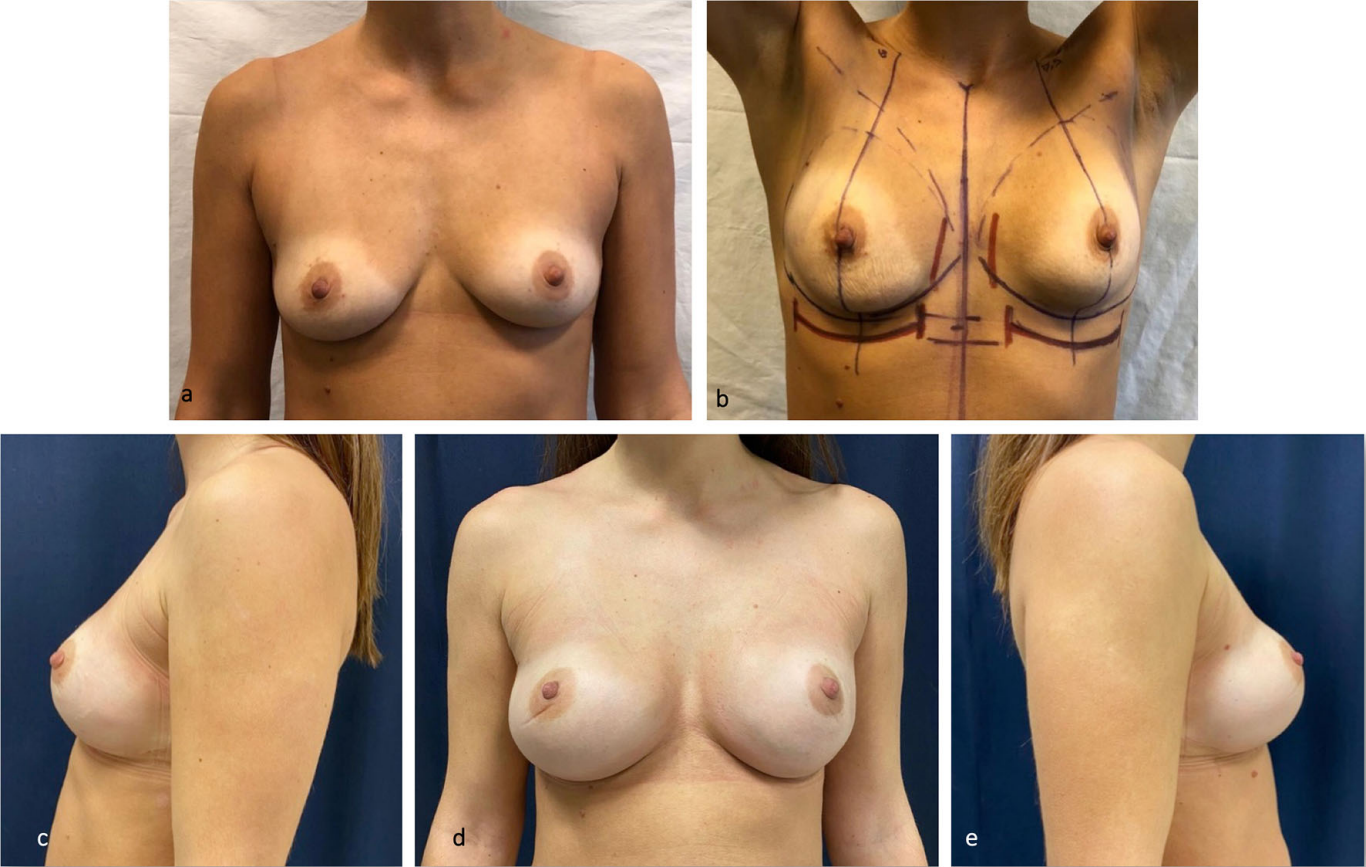
Patient who underwent bilateral subcutaneous mastectomy and direct-to-implant reconstruction with an increase in breast size. Preoperative frontal view (**a**). Markings of the planned position of the new IMFs (**b**). Post-operative result (**c**, **d**, **e**). The liposuction of the new, more caudal IMF was performed after mastectomy and implant positioning. The post-operative IMFs look natural and well defined

**Fig. 8:**
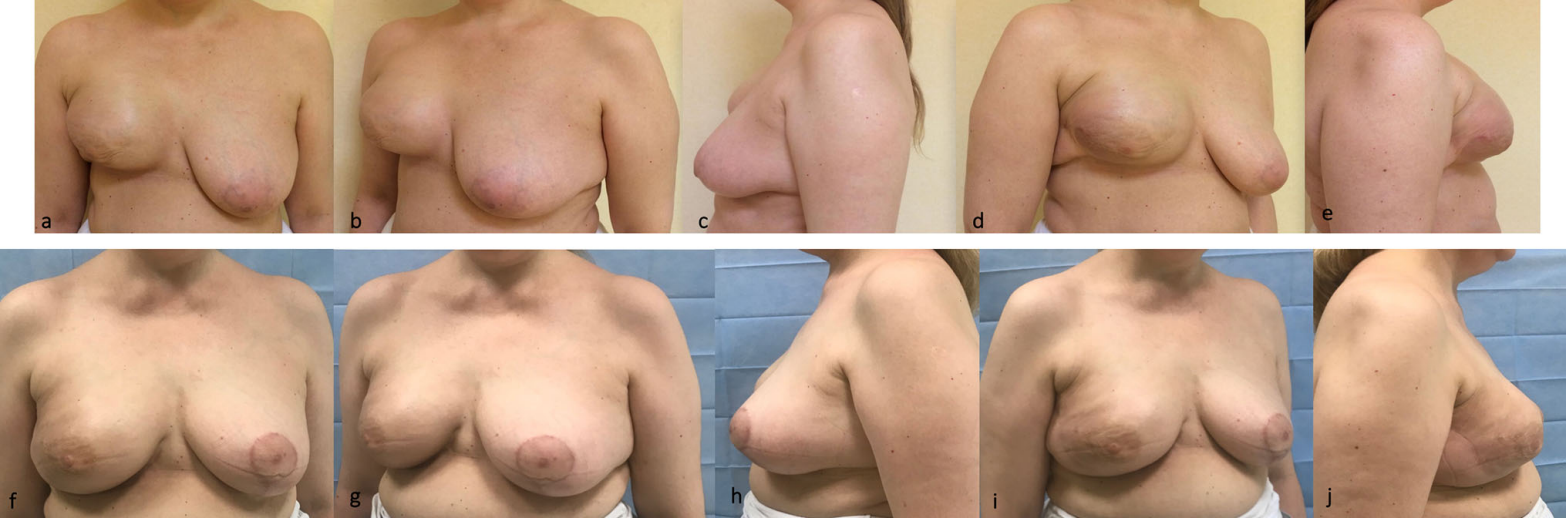
Unsatisfactory results after breast expansion due to previous quadrantectomy and radiotherapy. The lower pole is missing and the right IMF is more cranial then the left one (**a**, **b**, **c**, **d**, **e**). Good right breast ptosis and IMF definition have been achieved by recruiting skin for the lower pole by reverse abdominoplasty and stitches positioning (see text). Subdermal liposuction has contributed to optimize IMF’s definition. Breast reduction has been performed on the left (**f**, **g**, **h**, **i**, **l**).

A few clinical cases are shown in Figs. 6, 7, 8.

The follow-up of our patients extends from 6 to 64 months, with a mean of 28 months. Judging from our results (subjective opinion of patients and objective opinion of the evaluators), the IMF definition obtained with liposuction alone was well maintained over the years.

## Discussion

Breast reconstruction, to be successful, needs to restore the natural appearance of the breast and as symmetrical as possible to the contralateral side.

Recreating an accurate position and definition of the inframammary fold is an important part of the surgical treatment. Mastectomy pattern, breast size and breast shape, skin elasticity, and body habitus are only a few of the variables that can significantly influence the selection of the surgical approach and the final results of the IMF reconstruction. According to accurate anatomical studies, IMF does not contain adipose tissue, being a dermal structure, formed by a dense collagen network, held in place by a specialized superficial fascial system [11].

The IMF is frequently violated during mastectomy either for true oncologic needs or, more often, because of an underestimation by the surgeon of the IMF aesthetic importance as an anatomical landmark.

Despite the increasing use of skin-sparing techniques, the IMF is sometimes damaged and its reconstruction becomes essential to improve the natural appearance of the operated breast.

The absence or loss of definition of the IMF may jeopardize the result of an otherwise accurate reconstruction of the breast’s shape and size. This might be particularly evident in patients with unilateral breast reconstruction if procedures to improve symmetry such as mastopexy, breast reduction, and breast augmentation are not performed on the contralateral breast.

Over the years, several authors have described different surgical techniques, including the methods of Pennisi [12] and Ryan [13] (direct skin incision at the fold and dehepitelized flap sutured to the chest wall), the adipofascial flaps [2, 14] (like the pioneering use of an abdominal skin anchored to the chest wall by Versaci [15]), the use of multiple capsular flaps (such as the re-anchoring to the thorax of the posterior sheet of the expander capsule) [16] of internal sutures (as proposed by Nava [6]), of percutaneously placed stitches [5, 17] through minimal incisions [18]. All these techniques, although considered successful by the authors, are time-consuming and need practice and expertise to be optimized.

The use of capsular flaps or adipofascial flaps represents a good alternative. However, in some cases, we observed increased bleeding and a difficulty in deepening the fold without adding volume or reducing the size of the implant pouch. Sometimes the lower pole of the implant can move away from the skin envelope, causing an unsatisfactory breast ptosis.

Among other techniques that have recently been reviewed [4] are the use of barbed suture [8], attached medially to the costal cartilage [19], and the reconstruction of the IFM with acellular dermal matrices. The latter seems promising, but only one retrospective study has been published on its use [20].

Before adopting liposuction, we used to redefine the IMF, at the surgical time of exchange of the expander with implant, with internal stitches between the dermis and the deep tissues, similarly to the technique described by Nava [6]. The technique is effective but can be time-consuming, because it often requires several attempts before the desired result is obtained. Another drawback is that it sometimes creates indentations on the skin that last for several weeks or months and that sometimes require surgical revision and stitches removal [2]. We therefore reserved this technique to the cases in which the IMF was entirely missing, and not only suboptimally defined.

Several years ago, inspired by a brief article by Pinnella [7], we started to use the subdermal liposuction technique to recreate a natural, thin, adherent inframammary fold that imitates the normal anatomy with favourable cosmetic outcomes.

Although the use of liposuction in breast surgery is common, it is usually applied to obtain volume reduction in gynecomastia, macromastia, and ammoplasty [21–23] or lateral pole definition or lower pole skin re-draping like in Hall-Findlay superomedial breast reduction [24]. In a study reported by Maia and Pinto [25], the authors mention the use of liposuction/lipotunnelization to decrease breast volume, to decrease pillar height, and to recreate a new and well-shaped inframammary fold. The first, brief, communication that suggests the application to IMF definition in breast reconstruction and its potential advantages was authored by Pinnella in 1989 [7].

We believe that the IMF defining liposuction technique is effective because it recreates all the anatomical characteristics of the natural IMF. In fact, liposuction removes the adipose layer, thins the new fold, and produces a subcutaneous scarring that ties the dermis to the fascial layer, providing a natural-looking, well-defined fold.

Subdermal liposuction is easier and faster than any of the suture techniques [4], and avoids their complications, such as discomfort, pain, palpability of the stitches, foreign body granulomas, or dermal indentations [2].

**Fig. 9:**
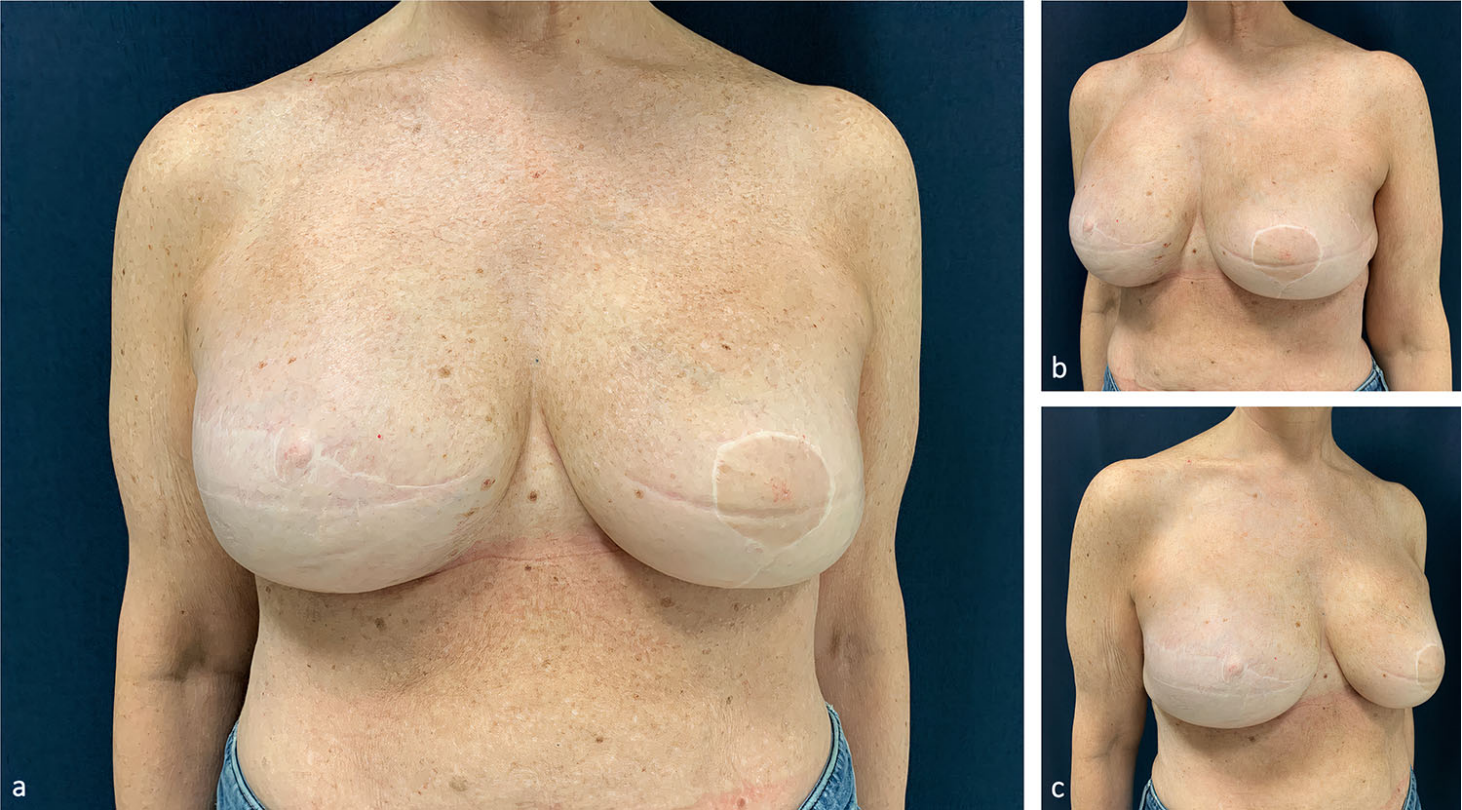
Follow-up result at 5 years in a patient who underwent right mastectomy, immediate reconstruction with expander, adjuvant radiotherapy, exchange of expander with implant, and the use of the liposuction technique alone to define IMF. On the left side, a breast reduction was performed (**a**, **b**, **c**).

Some concerns have been raised on the safety of liposuction in irradiated patients, particularly in the presence of radiodermitis or soft tissues hardening that may cause complications as severe as a pneumothorax. We performed liposuction uneventfully in 12 patients who had previously undergone radiotherapy, without developing cutaneous complications (Fig. 9).

Moreover, for liposuction we use a blunt cannula (3 or 4mm) that is positioned in a subdermal superficial plane, tangential to the thoracic cavity. With these precautions, the risk of causing a pneumothorax is very low, even in very thin patients.

If patients had previously undergone radiotherapy and have clinically evident soft tissue damage (oedema, erythema, thickening of dermal layer, hardening of the tissues, iperpigmentation or necrosis), we tend to avoid implant-based reconstruction and offer, whenever possible, autologous breast reconstruction. IMF definition would not be possible with liposuction alone in these patients.

**Fig. 10:**
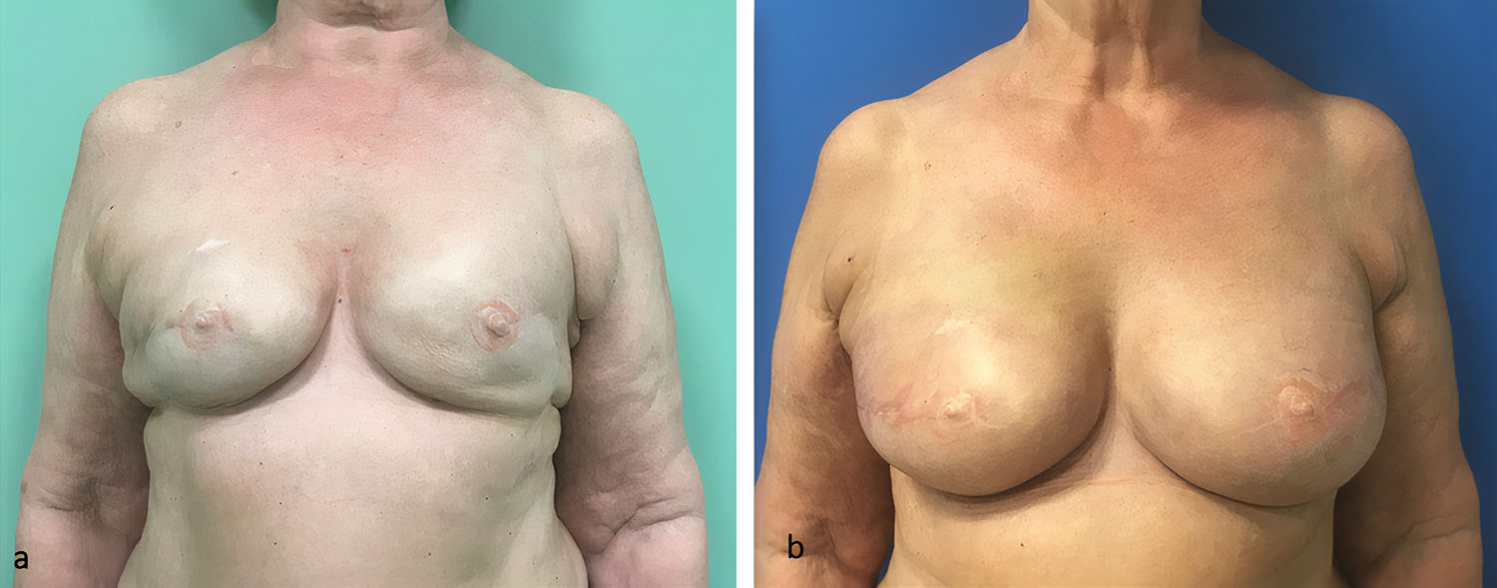
Patient with a BMI of 33 who underwent bilateral mastectomy, immediate reconstruction with expanders and adjuvant radiotherapy. Frontal view at the end of expansion and after radiotherapy (**a**). Post-operative result after exchange of expanders with implants and the use of the IMF liposuction technique alone (**b**). The post-operative IMFs look natural and well defined

Also, in women with high BMI we rarely use the IMF liposuction technique because we usually recommend autologous breast reconstruction with DIEP flap. If, in these patients, we use an implant-based reconstruction and decide to apply the IMF liposuction technique, we aspirate a thicker layer of subcutaneous tissue (Fig. 10) and, if needed, also consider the use of fold stitches to obtain an optimal IMF definition.

If the IMF is completely dislocated cranially or liposuction alone is not sufficient to optimize its shape, traditional internal stitches, which anchor the Scarpa’s fascia to the inferior border of the capsule, can be necessary to create a new IMF. We, however, always add liposuction to achieve a more natural appearing IMF.

In our series, these cases were a minority (12 over 88 breasts) and were usually a consequence of a more invasive mastectomy because of the disease characteristics or of the anatomical phenotype.

Based on our long-term satisfactory results, we suggest that the technique of subdermal liposuction is effective, easy to perform, minimally invasive, and long-lasting. It may be used alone or as an addition to traditional techniques.

## Conclusions

To define the IFM in breast reconstruction, we obtained satisfactory and long-lasting aesthetic results with the technique of dermal liposuction. The main advantage of the proposed technique is to restore the natural anatomical structure of the mammary crease, avoiding the pinched aspect often caused by the use of traditional internal sutures. Dermal liposuction is a safe, easily reproducible, and long-lasting procedure that does not create additional visible scars and produces a symmetric mammary crease also in overweight patients. When severe asymmetries are present, the technique can be combined with traditional stitching techniques.
